# Reinforcing Interdisciplinary Collaborations to Unravel the Astrocyte “Calcium Code”

**DOI:** 10.1007/s12031-022-02006-w

**Published:** 2022-05-11

**Authors:** Ana Covelo, Anaïs Badoual, Audrey Denizot

**Affiliations:** 1 Institut National de la Santé et de la Recherche Médicale (INSERM), U1215, NeuroCentre Magendie, 33077 Bordeaux, France; 2 University of Bordeaux, Bordeaux, 33077, France; 3 SERPICO Project-Team, Inria Centre Rennes-Bretagne Atlantique, Rennes Cedex, 35042 France; 4 SERPICO/STED Team, UMR144 CNRS Institut Curie, PSL Research University, Sorbonne Universités, Paris, 75005 France; 5 Computational Neuroscience Unit, Okinawa Institute of Science and Technology, Onna, 904-0495 Japan

**Keywords:** Astrocyte, Glia, Calcium, Interdisciplinary

## Abstract

In this review article, we present the major insights from and challenges faced in the acquisition, analysis and modeling of astrocyte calcium activity, aiming at bridging the gap between those fields to crack the complex astrocyte “Calcium Code”. We then propose strategies to reinforce interdisciplinary collaborative projects to unravel astrocyte function in health and disease.

## Introduction

**Fig. 1:**
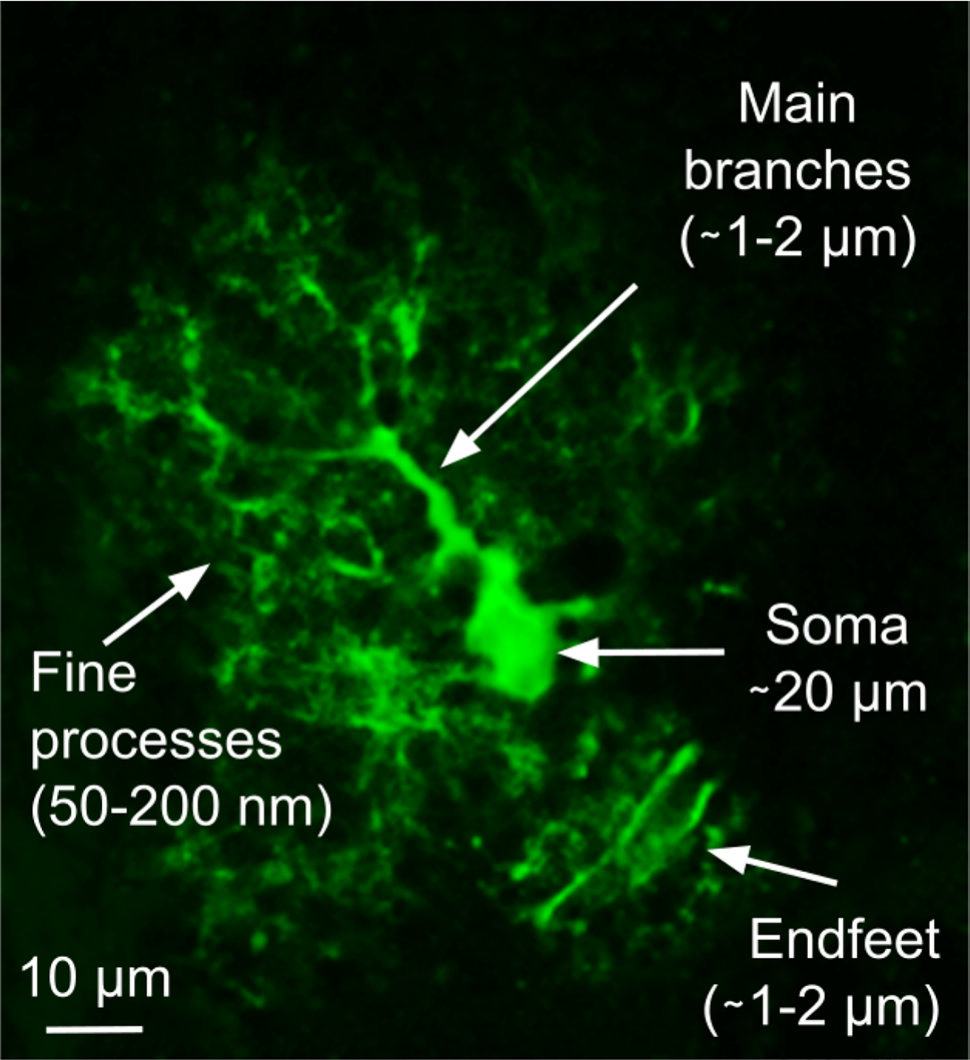
Confocal image of an astrocyte expressing GCaMP6f (maximum intensity projection over time) that shows its different structural compartments and their size

Astrocytes, the most abundant non-neuronal cells of the nervous system, are essential to brain function, from synaptogenesis and neurotransmission to higher brain functions such as memory and learning (Verkhratsky and Nedergaard 2018). Those functions of astrocytes are altered in various brain diseases such as epilepsy, brain tumors, neurodegenerative diseases, Down syndrome, major depressive disorder and schizophrenia (Verkhratsky and Nedergaard 2018). Astrocytes notably respond to stimuli with transient elevations in cytosolic calcium concentration, referred to as calcium signals. Those calcium signals are essential to brain function and are altered in various brain diseases (Verkhratsky and Nedergaard 2018; Semyanov et al. 2020). Importantly, astrocyte calcium signals can trigger the release of molecules referred to as gliotransmitters that modulate neuronal communication at synapses (for recent reviews on gliotransmission and the associated controversies, see (Savtchouk and Volterra 2018; Fiacco and McCarthy 2018)). Better understanding astrocyte physiology and the communication between astrocytes and other cells of the central nervous system thus rely on our ability to make sense of those calcium signals. Astrocyte calcium signals are characterized by diverse spatial (from microdomains to signals spreading within astrocyte networks) and temporal characteristics (from hundreds of milliseconds to tens of seconds) (Semyanov et al. 2020). The majority of those signals occur in fine astrocyte compartments (50-200 nm), referred to as processes, that account for as much as 80 % of the volume of an astrocyte, yet cannot be resolved by diffraction-limited light microscopy (Semyanov et al. 2020; Bindocci et al. 2017) (see Fig. 1). This strongly hinders our ability to characterize astrocyte calcium activity, from the molecular pathways involved to the quantification of the spatio-temporal properties of the signals. Consequently, the functions of the various signals observed, referred to as the astrocyte “Calcium Code”, remain unclear. Better characterizing astrocyte activity concomitantly with the activity of other brain cells will be essential to unravel the roles of astrocyte calcium signals in brain function (Adamsky et al. 2018). Please refer to the review (Semyanov et al. 2020) for more details on the current challenges associated with the study of calcium signals in astrocytes.

In this review article, we highlight the importance of reinforcing interdisciplinary collaborations to crack the astrocyte “Calcium Code”, with a focus on the characterization of the properties of astrocyte calcium signals. We present the major insights from and challenges faced in data acquisition, analysis and modeling of astrocyte calcium activity and propose strategies to facilitate and strengthen collaborations between these fields, which are essential to unravel the functions of astrocyte calcium signals in health and disease.

## Acquisition of Astrocyte Calcium Signals

Data acquisition is the first step to characterize astrocyte calcium activity. In this section, we present a brief overview of the tools that are available for imaging astrocyte calcium signals, both in slices and in vivo. We further highlight the insights, challenges and perspectives associated with measuring calcium signals in astrocytes.

### Imaging Tools for Astrocyte Calcium Acquisition

The development of calcium indicators, which change their fluorescence properties when binding to calcium ions, allowed neuroscientists to study astrocyte calcium activity. Numerous indicators exist, characterized by diverse kinetics and diffusion properties, so that they should be chosen carefully. In the early days, chemical calcium dyes, such as Fluo-4 or Oregon Green BAPTA, were commonly used (Fellin et al. 2004; Fiacco and McCarthy 2004; Hirase et al. 2004; Parri et al. 2001; Perea and Araque 2005; Serrano et al. 2006). One of the main caveats of these chemical sensors is the low signal-to-background noise ratio of the resulting signals, which only allows visualizing calcium signals in the soma and the main thick branches of astrocytes (see Fig. 1), unless loaded through a patch-clamp recording pipette and visualized with high-resolution microscopy (Di Castro et al. 2011; Panatier et al. 2011; Min and Nevian 2012). More recently, the development of genetically encoded calcium indicators (GECIs) (Berlin et al. 2015) has considerably improved our understanding of astrocyte calcium dynamics. Various GECIs have been developed in the last years that can be imaged by different tools, for precise or wide imaging at cellular or subcellular levels (Agarwal et al. 2017; Durkee and Araque 2019; Shigetomi et al. 2013; Shigetomi et al. 2010; Serrat et al. 2021). GECIs have several advantages compared to classical calcium dyes. First, they can be easily targeted to be expressed specifically in astrocytes. Moreover, they provide a higher signal-to-background noise ratio compared to classical calcium dyes and diffuse better into the fine processes. Additionally, GECIs can be expressed in live organisms, thus allowing in vivo calcium imaging in anesthetized (Lines et al. 2020; Poskanzer and Yuste 2016; Stobart et al. 2018; Serrat et al. 2021), awake head-fixed (Agarwal et al. 2017; Paukert et al. 2014; Srinivasan et al. 2016; Stobart et al. 2018) or freely moving mice during consecutive behavioral sessions (Corkrum et al. 2020; Paukert et al. 2014; Qin et al. 2020). While many GECIs have been designed in the last years for neurons, only a few are available to target astrocytes specifically (reviewed in (Lohr et al. 2021)). These GECIs have different spectral, temporal and spatial properties that make them suitable for specific experimental applications (Tong et al. 2013). Importantly, they yield calcium signals with different spatio-temporal properties that may not be comparable and may be difficult to analyze with certain software (see “Analysis of Astrocyte Calcium Signals”).

Astrocytes display most of their activity in their fine processes. The majority of those signals are spatially restricted, forming so-called microdomains, and display strikingly diverse spatio-temporal properties (Khakh and McCarthy 2015). Understanding the physiological relevance of those calcium signals requires powerful imaging techniques that can be used in combination with complementary methods to manipulate astrocyte and neuronal activity, such as electrophysiology, optogenetics, pharmacology and behavioral tests. Notably, because of the small size of astrocyte processes, *high-resolution microscopy* is needed to obtain a thorough view of the astrocyte calcium activity. Both confocal and two-photon microscopy are good options for imaging astrocyte calcium activity because these setups are generally compatible with other techniques, allowing for the study of calcium signals at the tripartite synapse level in slices and in vivo in anesthetized (Poskanzer and Yuste 2016; Stobart et al. 2018; Lines et al. 2020; Serrat et al. 2021) or awake head-fixed mice (Paukert et al. 2014; Agarwal et al. 2017; Stobart et al. 2018; Srinivasan et al. 2016). Light sheet fluorescence microscopy (LSFM) and Lattice LSFM are novel imaging techniques that allow fast 3D scanning with low phototoxicity and a resolution comparable to confocal microscopy (Chen et al. 2014; Ducros et al. 2019). Therefore, those techniques are excellent imaging options for experiments in brain slices. Please refer to Table 1 for an overview of optical resolution, phototoxicity/photobleaching, and compatibility of the different imaging techniques. *High-resolution microscopy* allows recording calcium signals at a high acquisition speed (in the order of ms), but its spatial resolution is limited by diffraction (x-y: 0.2–0.3 $\mu$m and z: 0.5 $\mu$m at best) and, thus, does not allow visualizing fine processes in detail. Recent studies have used *super-resolution microscopy* such as stimulated emission depletion (STED) and stochastic optical reconstruction microscopy (STORM) to study astrocyte morphology at the tripartite synapse level in live tissue (Panatier et al. 2014; Heller et al. 2020; Arizono et al. 2020). STED microscopy has revealed that the complex spongiform morphology of astrocyte processes contains functionally isolated nanostructures that are characterized by spatially restricted calcium signals (Arizono et al. 2020). Currently, because of their low acquisition speed and high laser intensity, which induces high photobleaching of calcium sensors, *super-resolution microscopy* techniques cannot be used to perform calcium imaging. Thus, in the aforementioned study, calcium signals were acquired using *high-resolution microscopy* and were then mapped onto super-resolution structural images. Super-resolution imaging requires powerful computational tools, both for acquisition and analysis, which are not broadly available in the experimental community (in terms of knowledge, software and hardware) and emphasizes the need to establish collaborations between experimental and computer scientists.

**Table 1:** Overview of the main calcium imaging techniques used to study astrocyte calcium signals

**Imaging method**	**Optical resolution**	**Photobleaching & phototoxicity**	**Preparation**	Compatible with fast 3D scanning^*^	**Compatible with other techniques**
Wide-field	Soma & main branches	High	In vitro & in vivo (anesthetised & head-fixed)	No	Electrophysiology, pharmacology, wide-field photostimulation
Confocal	Soma, main branches & fine processes	High	In vitro & in vivo (anesthetised & head-fixed)	No	Electrophysiology, pharmacology, localized photostimulation
Two-photon	Soma, main branches & fine processes	^**^Low	In vitro & in vivo (anesthetised & head-fixed)	Yes, depending on the microscope	Electrophysiology, pharmacology, localized photostimulation
LSFM	Soma, main branches & fine processes	Low	^***^In vitro	Yes, faster than two-photon	Electrophysiology, pharmacology
Lattice LSFM	Soma, main branches & fine processes	Very low	^***^In vitro	Yes, faster than LSFM	Electrophysiology, pharmacology
Fiber photometry	Population	High	In vivo (freely behaving)	No	Electrophysiology, wide-field photostimulation
Miniscopes	Soma	High	In vivo (freely behaving)	No	Wide-field photostimulation

^*^The ability to perform 3D fast scanning depends on the scanning method that the microscope uses, which varies depending on its hardware settings; ^**^Photobleaching and phototoxicity can be high at the focal plane with two-photon microscopy because it uses high intensity lasers, but it is low if the whole sample is considered (see (Benninger and Piston 2013)); ^***^Note that Light sheet fluorescence microscopy (LSFM) and Lattice LSFM cannot be used in vivo in postnatal murine models but can be used in vivo in embryos

### The Need for Interdisciplinary Approaches

It is important to keep in mind that experimental approaches have inherent limitations. First, calcium indicators are calcium buffers. Therefore, calcium indicators compete with calcium binding sites in the cell, altering calcium signals and the normal functioning of the cell. Second, the spatial and temporal characteristics of the measured signals are constrained by the imaging technique as well as the kinetics of the calcium indicator used. It is thus possible that some faster or smaller calcium signals than those currently reported exist in astrocytes that cannot be detected by the calcium imaging tools that are currently available. Importantly, this effect can be amplified during 3D scanning for calcium signals that are faster than the z-scanning time. Lastly, experimental manipulations, such as using a knock-out mouse line or bath applying drugs, can have unexpected off-site effects that can impact the results, making it difficult to extract causal relationships between the experimental manipulation and the obtained results. Collaborative work with computational scientists is essential to build mechanistic models to go beyond those limitations. For example, models have been essential to characterize the effect of the concentration, kinetics and diffusion coefficient of calcium buffers, such as calcium indicators, on calcium dynamics (Matthews and Dietrich 2015; Schwaller 2010). Models can thus be used to predict the free calcium signals that would occur in the absence of indicators. Further, models can measure *in silico* calcium signals at very high spatial and temporal resolution (depending on the method used, see "Modeling Astrocyte Calcium Signals"), thus predicting the range of calcium signals that could not be resolved experimentally.

## Analysis of Astrocyte Calcium Signals

In the quest of characterizing astrocyte calcium signals, the key role of analysis is to provide tools to experimentalists and modelers to process their data, of increasing size and complexity. Statistical as well as advanced computational image analysis tools are thus needed. In this section, we focus on the analysis of calcium images, which is meant to quantify what is observed, *i.e.*, to extract meaningful information or measurements from images. In particular, we emphasize the importance of developing computational image analysis tools dedicated to the quantification of astrocyte calcium signals, and the challenges to get there.

### Image Analysis to Characterize Astrocyte Calcium Signals

Decoding the astrocyte “Calcium Code” involves the characterization of the spatio-temporal dynamics of astrocyte calcium signals. Computational image analysis tools aim at accurately detecting all astrocyte calcium signals in a sequence of microscopy images and, for each signal, extracting its dynamical and spatial features, such as its amplitude, its duration, its trajectory, its propagation speed, the location from which it originates and its volume. Various information, such as the number of calcium signals in a specified region or cell, their frequency at a position, and the different types of signals induced by a stimulus, can be deduced from those measurements.

From an image analysis point of view, reaching this ideal of output information requires preprocessing steps (*e.g.*, denoising, deconvolution, motion correction) as well as the detection, the segmentation and the quantification of the calcium signals in a sequence of microscopy images, which is very challenging due to the complex nature of these signals. First, calcium signals are characterized by various durations (from milliseconds to tens of seconds), frequencies and signal-to-noise ratios. Second, their spatial spreads vary from microdomains to signals that propagate within the astrocyte in regions of various sizes and shapes. Third, they can overlap in space and time (Srinivasan et al. 2015). As most signals occur in fine astrocyte processes that cannot be fully resolved by diffraction-limited light microscopy techniques, image analysis methods cannot rely on morphological criteria to detect calcium signals, which also complexifies their quantification. In addition, the developed image analysis methods should ideally operate across data with different spatial scales, taken in vivo or in vitro, and acquired with different imaging techniques.

### Lack of Computational Image Analysis Tools Adapted to the Complexity and Diversity of the Data

Recently, several image analysis algorithms have been developed to quantify astrocyte calcium signals in 2D+time microscopy images. Among them, we can cite GECIquant (Venugopal et al. 2019), CaSCaDe (Agarwal et al. 2017), FASP (Wang et al. 2017), AQuA (Wang et al. 2019) and, more recently, Begonia (Bjørnstad et al. 2021) and Astral (Dzyubenko et al. 2021). Most of these methods are ROI-based approaches (ROI: region of interest), meaning that calcium signals are analyzed through fixed spatial boundaries in the image. As the spatial spread of calcium signals can vary over time and become larger than or get out of the ROI, those approaches can lead to inaccurate or partial detection of the signals. To solve this issue, event-based algorithms have been developed, such as AQuA (Wang et al. 2019). For more details about these algorithms (*e.g.*, analysis approach and outputs), please refer to the dedicated section in the review article from (Lia et al. 2021).

The aforementioned analysis tools have considerably improved the detection and characterization of astrocyte calcium signals. Yet, their use can be restricted, either because they are not adapted to the diversity of acquisition modes and calcium indicators (see “Acquisition of Astrocyte Calcium Signals”) or because they are not open-source or not user-friendly (Carpenter et al. 2012). This can constrain some neuroscientists to implement “in-house” analysis pipelines, which is time-consuming and less reproducible, or to use tools that were initially developed for neuronal calcium imaging analysis, such as CaImAn (Giovannucci et al. 2019), Suite2P (Pachitariu et al. 2017) and LC_Pro (Francis et al. 2012). This latter approach is not optimal as astrocytes differ from neurons in many ways. For example, they have a different morphology. Further, notably because astrocytes are not polarized cells, their calcium signals display different spatio-temporal properties than the ones of neurons.

The continuous scientific and technical advances in calcium imaging will always call for new and adapted image analysis algorithms. Until now, most of the quantification of calcium signals has been performed on 2D+time fluorescence microscopy data. The recent emergence of 3D+time imaging techniques gives access to new and major structural and dynamical information, such as the number of calcium signals occurring in an entire astrocyte volume, their synchronization, their trajectory and the location from which they originate (Bindocci et al. 2017). To the best of our knowledge, there is currently no image analysis tool to detect, segment and quantify astrocyte calcium signals in 3D+time microscopy images.

### Challenges Hindering the Development of 3D+time Image Analysis Tools

The main reason why the quantification of astrocyte calcium signals has been so far restricted to 2D+time images is because of the trade-off between temporal and spatial resolution in microscopy techniques. The access to a refined 3D imaging of the dynamical behavior of calcium signals in astrocytes is quite recent, owing to the emergence of microscopes enabling a high 3D spatio-temporal resolution with low phototoxicity (*e.g.*, lattice light sheet fluorescence microscopy (LSFM) (Chen et al. 2014; Ducros et al. 2019) and of genetically encoded calcium indicators (GECIs) (Berlin et al. 2015). Despite these scientific and technical advances, the development of 3D+time image analysis tools tailored for the astrocyte calcium activity is not straightforward and calls for new quantitative analysis algorithms with new constraints and challenges. First, a key challenge in the development of 3D+time image analysis tools is the memory and computational costs required to process large 3D+time data. To give the reader an idea, the equivalent of one hour of acquisition of Lattice LSFM data represents about 1 To of data. Importantly, the developed analysis tools should be accessible and thus ideally be able to run on standard desktop computers. To tackle this challenge, ingenious solutions for image processing are needed such as using data-dimensionality reduction techniques. Second, and more critical, reliable and large amounts of labeled data are not available. Such datasets are crucial to evaluate image analysis tools and to train data-driven tools, which are increasingly common with the emergence of deep learning in biological image analysis (Meijering 2020). Manually annotating 3D time-lapse images is a tedious task—mainly because of the complex visualization in 4D space—that cannot be performed reliably. There is a significant intra- and inter-experimenter variability. There is currently a major lack of annotations of astrocyte calcium activity images. Note that this is also true for other datasets of 3D images in live tissue (Yakimovich et al. 2021). For all of those reasons, a common and promising approach is to use realistic synthetic datasets with known ground-truths (*i.e.*, all morphological and dynamical properties are known and controlled) to train and quantitatively assess the performance of analysis software. This highlights the need for developing models and simulators that are able to mimic real image sequences.

### Need for Public Realistic Synthetic Datasets: Join the Forces!

To solve the difficulty to reliably label calcium signals in microscopy images, a promising approach consists in generating 3D+time synthetic datasets that realistically depict astrocyte calcium signals observed in real microscopy images. To be as realistic as possible, the simulation should be driven by a biophysical model that describes the calcium signals at the nanoscale, which requires close collaboration between image analysts, modelers (see “Modeling Astrocyte Calcium Signals”) and experimentalists (see “Acquisition of Astrocyte Calcium Signals”). For instance, a recent interdisciplinary collaboration (Badoual et al. 2021) has resulted in the creation of a simulator to generate realistic sequences of 3D lattice LSFM images depicting calcium activity in the sponge-like network of astrocyte processes by integrating a simplified version of the kinetic model developed by (Denizot et al. 2019). In addition to hopefully opening the door to the deployment of 3D+time image analysis tools to quantify astrocyte calcium activity, these simulators could also help modelers tuning their models and the parameters in a faster way than using computational simulations, which are often time and computationally expensive. A major challenge to develop such simulators is the complexity of evaluating the similarity between the generated synthetic images and real images. Implementing rigorous methods to evaluate synthetic astrocyte calcium images will thus be essential to ensure the success of this approach. Note that these synthetic data are essential to guide analysts in the development of their algorithms, but final qualitative validation on real images is still required.

## Modeling Astrocyte Calcium Signals

Models correspond to simplifications that describe relevant parameters of a system of interest (its elements, their states and their interactions), allowing for better quantification, visualization, and understanding of the system. The famous quotation from George Box, “All models are wrong but some are useful” (Box 1980), highlights that models are incomplete representations of the system as a whole, yet provide crucial insights into the system’s behavior and dynamics. Such insights would not be grasped by a model as complex as the system of interest itself.

Depending on the question and hypothesis that emerge from experimental data, modelers choose different approaches and toolkits (see Table 2). For example, models studying calcium activity in microdomains will need a higher spatial resolution than models of somatic signals. Further, the modeling approaches that are well-suited to study calcium microdomains, such as particle-based methods (see (Denizot et al. 2020) for a review), are more accurate but extremely demanding in terms of computational power and simulation time. Simulating hundreds of seconds of calcium activity in a fine astrocyte process (*e.g.*, 1 $\mu$m long, 200 nm in radius) can take days to compute, so that using those tools to simulate signals in a whole cell or in a network of cells is currently unfeasible. Please note that the computation time to simulate, *e.g.*, 1 millisecond of calcium activity varies not only depending on the modeling technique used, but also on the computational resources available in each laboratory, on the volume and number of reactions modeled as well as the simulation time. To learn more about the different approaches that can be used to model reactions, their insights and limitations, please refer to dedicated reviews (Burrage et al. 2011; Denizot et al. 2020). The goal of this section is not to present an exhaustive list of astrocyte models (see (Denizot et al. 2020; Manninen et al. 2018; Oschmann et al. 2017)), to review existing models of calcium signaling (reviewed in (Dupont et al. 2011; Dupont et al. 2016; Dupont and Croisier 2010; Dupont and Sneyd 2017; Rüdiger 2014; Rüdiger and Shuai 2019)), or to present a detailed list of modeling tools to model calcium signals (Dupont et al. 2016; Blackwell 2013). Rather, we emphasize the key insights that can be gained from models of astrocyte calcium activity as well as the challenges that computational neuroscientists are currently facing.

### Insights from Modeling into Biological Processes

Mathematical and computational models are powerful tools that provide new insights in the mechanisms that regulate calcium activity in astrocytes and generate testable predictions. First, models can be used to conduct *in silico* experiments that are time-consuming or unfeasible experimentally. Models have for example been used to finely tune the spatial distribution of calcium channels (molecules that, when open, result in a calcium influx into the cytosol, forming a calcium signal) within the cell and explore its impact on astrocyte activity (Denizot et al. 2019; Savtchenko et al. 2018). Moreover, models can be used to generate realistic datasets that can be used to train tools developed to characterize the system’s behavior (see “Analysis of Astrocyte Calcium Signals”) (Badoual et al. 2021). Lastly, computational models are useful to go beyond correlational observations and to propose mechanistic principles that explain experimentally observed data. For example, models have shown the effect of cellular morphology on the compartmentalization of calcium signals in dendritic spines (Bell et al. 2019; Biess et al. 2011; Santamaria et al. 2011; Yasuda 2017) as well as in astrocyte processes (Denizot et al. 2022). For a recent review on the insights gained from computational approaches on astrocyte function as well as strategies to start incorporating astrocyte calcium signals in systems neuroscience to better understand how astrocytes contribute to brain computation, see (Kastanenka et al. 2019). Overall, modeling approaches can provide key insights to astrocyte physiology.

**Table 2:** Brief summary of the main modeling approaches that are commonly used to model astrocyte calcium activity, their insights, limitations and examples. Biological processes are inherently noisy. When the system that is modeled contains a large number of molecules, this noise can be averaged. Such models are called deterministic and describe the variation of molecular concentrations over time. They are often used to describe calcium signals at the whole cell and at the network levels. When the system of interest contains a small number of molecules or ions, typically small subcellular compartments like astrocyte processes, this approximation is no longer valid and the stochastic nature of molecular reactions has to be taken into account in the model. Further, models can be spatial, *i.e.* take into account the position and potential diffusion of molecules in the cell, or well-mixed, *i.e.* at each time step, any molecule can virtually move anywhere in the cell. The location of the molecules and cell morphology is thus not taken into account in well-mixed models

**Name of the modeling approach**	**Spatial**	**Describe variation of concentration**	**Tracks individual molecules**	**Computational cost**	**Common use**	**Examples**
Well-mixed, deterministic	No	Yes	No	Very low	Astrocyte network/whole cell	(Lavrentovich and Hemkin 2008; Oschmann et al. 2017; De Pittá et al. 2009)
Well-mixed, stochastic	No	Yes	No	Low	Astrocyte network/whole cell	(De Pittá et al. 2016; Kuchibhotla et al. 2009; Riera et al. 2011)
Spatial, deterministic	Yes	Yes	No	Low-intermediate	Whole cell/Signal propagation in major branches	(Brazhe et al. 2018; Cresswel-Clay et al. 2018; Höfer et al. 2002; Lallouette et al. 2014; Postnov et al. 2009; Savtchenko et al. 2018; Gordleeva et al. 2018)
Spatial, stochastic	Yes	Yes^*^	Yes^**^	High	Spongiform domain	(Denizot et al. 2019; Héja and Kardos 2020)

Note that the characteristics presented in this table are indicative as the usage and computational cost of a given model vary greatly depending on the precise method implemented and the number of molecules/reactions modeled (see (Burrage et al. 2011; Denizot et al. 2020) for reviews)

^*^Calcium concentration in spatial stochastic simulations can be deducted from the number of molecules tracked and the system’s volume; ^**^Some spatial stochastic techniques track individual molecules (particle-based) while others track the number of molecules in small sub-compartments (voxel-based). See *e.g.* (Smith and Grima 2018) for a review

### Main Challenges Associated With the Development of Models of Astrocyte Calcium Activity

Computational neuroscientists are facing major challenges to build models of astrocyte calcium activity. First, a lot of data are currently missing or not shared publicly, so that most parameter values used in the astrocyte models that are currently available are derived from data obtained in other cell types. Those data include the concentration and sub-cellular distribution of endogenous buffers, the diffusion coefficient of diffusing molecules involved in calcium dynamics in astrocytes, the distribution of the major calcium channels and pumps in the cell, the dynamic remodeling of the morphology of the cell and of its internal calcium stores in live tissue. Second, the computational cost of simulations increases drastically as the accuracy of the model increases, so that a trade-off often has to be made by the modeler, resulting in models with few reactions or low spatial resolution. Further, some of the currently available models suffer from a lack of availability, replicability, and reproducibility (Manninen et al. 2018). Lastly, models often focus on specific spatio-temporal scales of astrocyte activity. Bridging those models together is critical to better understand the involvement of local calcium signals in higher-level brain functions such as cognition and learning. Building such multi-scale models is challenging but should provide unprecedented insights in the involvement of astrocyte calcium signals in the activity of neural circuits and overall in brain (dys-)function.

### Is There such a Thing as a Generic Astrocyte Model?

Although astrocytes share common morphological and biochemical characteristics, they are remarkably heterogeneous. The diversity of astrocyte morphology has been described as early as 100 years ago by Cajal and morphology-based classifications of astrocytes have been proposed (Emsley and Macklis 2006). Astrocyte electrophysiological properties (D’Ambrosio et al. 1998; Nimmerjahn et al. 2009; Takata and Hirase 2008), gene (Doyle et al. 2008; Molofsky et al. 2014; Shah et al. 2016) and protein expression levels (Oberheim et al. 2012) also vary drastically depending on the brain region under study. Those observations suggest that astrocytes are a heterogeneous cell population, questioning the specificities and roles of individual sub-types. For more details, see dedicated reviews on astrocyte heterogeneity (Bayraktar et al. 2015; Haim and Rowitch 2017; Verkhratsky and Nedergaard 2018; Zhou et al. 2019). Whether the diverse functions of astrocytes in the brain rely on molecularly and morphologically distinct sub-populations of astrocytes is still poorly understood, yet crucial to uncover the functions of astrocytes in the healthy and diseased brain. A recent study identified sub-populations of astrocytes that selectively contributed to specific functions such as synaptogenesis and tumor invasion of glioma (John Lin et al. 2017). Incorporating this diversity in astrocyte models by building models of specific sub-populations of astrocytes rather than the currently available generic astrocyte models will be essential to provide insights into the functional implications of the molecular and morphological heterogeneity of astrocytes that have been reported recently.

### Need for Interdisciplinary Collaborations to Improve Models of Astrocyte Calcium Activity

Several strategies and perspectives could be developed to go beyond the aforementioned challenges to model astrocyte calcium activity. First, computational neuroscientists would highly benefit from the existence of open-source datasets, which could be used to build and test models. Such datasets are crucial for data-driven modeling practices, which rely on strong iterative collaborative work between experimentalists and computational neuroscientists. Moreover, several good practices and step-by-step modeling guides have been published to ensure the reproducibility of models (Blohm et al. 2020; Kording et al. 2018; Novére et al. 2005). Lastly, initiatives such as the Neuromatch Academy courses and conferences (Achakulvisut et al. 2021; van Viegen et al. 2021) provide an unprecedented opportunity to build an accessible, democratic, inclusive, international and interdisciplinary community aiming at using computational approaches to improve our understanding of brain function.

## Perspectives

**Fig. 2:**
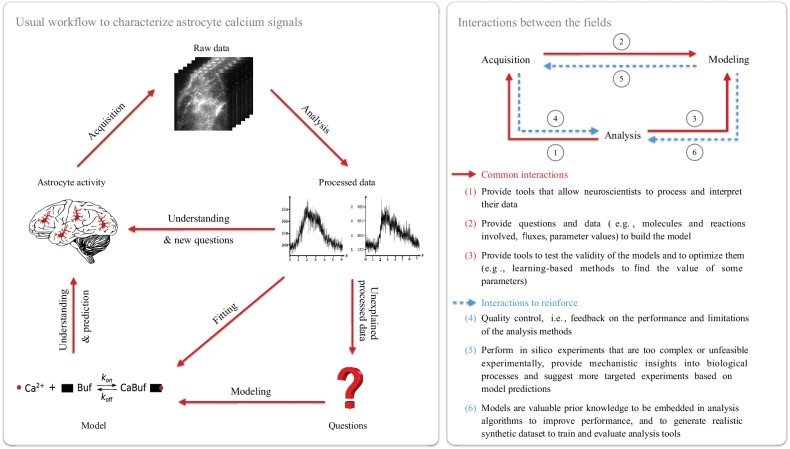
Reinforcing interdisciplinary collaborations to unravel the astrocyte “Calcium Code”. Left: workflow for the characterization of calcium signals involving the fields of acquisition, analysis and modeling. The raw data acquired by experimentalists include *e.g.*, calcium images, structural images or omics data. Raw data processing by analysts results in dynamical (*e.g.*, duration, trajectory, frequency) and structural characterization (*e.g.*, protein localization, cell morphology) of astrocytes as well as the quantification of protein expression levels, for example. Right: schematic representation of the interactions between the fields. Interactions to reinforce are highlighted in dashed blue lines (4, 5, 6)

Astrocytes are cells that display a highly complex activity that is essential to brain function. Characterizing the diverse signals displayed by active astrocytes and understanding their physiological roles, the “Calcium Code”, are the biggest challenges of the field and are crucial to understand the involvement of astrocytes in brain function. In this short review article, we highlighted the different insights that can be gained from each field that studies calcium signals in astrocytes and the major challenges that they are facing. Key challenges that prevent us from making sense of astrocyte calcium activity have arisen from our discussions during our interdisciplinary workshop, hosted by the $1^{st}$ Virtual Conference of the European Society for Neurochemistry “Future perspectives for European neurochemistry—a young scientist’s conference”, in May 2021, entitled “Let’s join forces—Bridging the gap between experimental, computational and data sciences to disentangle astrocyte calcium activity”. Those challenges include:

- The development of analysis tools allowing accurate detection and characterization of individual calcium signals in astrocytes are lacking, notably in 3D+time.
- There is no consensus in key definitions and terminology, which further hinders efficient communication across fields (*e.g.*, calcium microdomain/nanodomain, processes/leaflets, Calcium Code, gliapil/spongiform domain).
- A lot of data are missing to fully grasp the mechanisms regulating astrocyte calcium signals and their physiological roles. For example, local and regional variability of the expression levels of proteins involved in calcium signaling, both in health and disease, remain to be characterized. The morphology of perisynaptic astrocyte processes and their organelles, together with their dynamical remodeling, also remain to be uncovered in live tissue.
- Raw data are rarely shared in public repositories. Notably, labeled datasets are needed to evaluate image analysis tools and to train data-driven tools. Providing public access to such datasets has contributed to fast improvements in other fields, such as the development of tools detecting the onset of epileptic seizures (Brinkmann et al. 2016).
- Interdisciplinary events and projects are rare, which constitutes a major barrier to our efforts to unravel the astrocyte “Calcium Code”. Indeed, scientists from different fields lack opportunities to discuss, share ideas and knowledge. The interactions between fields working on astrocyte calcium signals and opportunities for improvements are highlighted in Fig. 2. We believe that such joint efforts are essential to fully grasp the complex properties and functions of astrocytes.

Reinforcing interdisciplinary projects, bringing together experts from different fields, will be crucial to ensure our success in cracking the astrocyte “Calcium Code”. Such collaborative projects are still rare in the field, which might result from the high fragmentation of research projects and fields working on astrocyte physiology, often presenting their work in different, highly specialized conferences and journals. We propose initiatives that will facilitate the emergence of new interdisciplinary projects:

- Agreement on shared definitions and terminology across fields.
- Sharing datasets, together with all the relevant information on the data acquisition, processing and modeling (if relevant) methods used. This might require the creation of an online platform to store and discuss data on astrocytes.
- Sharing user-friendly data analysis tools, including providing the code in open-access and the dataset(s) used to facilitate their dissemination to the whole community.
- Organization of recurrent meetings and events that bring together experts from various fields of expertise.

Because of the complexity of astrocyte morphology and signaling, interdisciplinary projects will be essential to not only crack the astrocyte “Calcium Code”, but also to successfully improve our understanding of astrocyte (patho-)physiology and to propose models of astrocyte function.

## Data Availability

Data sharing is not applicable to this article as no datasets were generated or analyzed during this study.
